# Design Rules for a Wearable Micro-Fabricated Piezo-Resistive Pressure Sensor

**DOI:** 10.3390/mi13060838

**Published:** 2022-05-27

**Authors:** Borzooye Jafarizadeh, Azmal Huda Chowdhury, Iman Khakpour, Nezih Pala, Chunlei Wang

**Affiliations:** 1 Department of Mechanical and Materials Engineering, Florida International University, Miami, FL 33174, USA; bjafa001@fiu.edu (B.J.); achow030@fiu.edu (A.H.C.); ikhak002@fiu.edu (I.K.); 2 Department of Electrical and Computer Engineering, Florida International University, Miami, FL 33174, USA; npala@fiu.edu; 3 Center for the Study of Matter at Extreme Conditions, Florida International University, Miami, FL 33199, USA

**Keywords:** piezo-resistive pressure sensor, micro-pyramid, flexible, arterial pulse, wearable heart rate monitoring

## Abstract

Wearable flexible piezo-resistive pressure sensors hold a wide-ranging potential in human health monitoring, electronic skin, robotic limbs, and other human–machine interfaces. Out of the most successful recent efforts for arterial pulse monitoring are sensors with micro-patterned conductive elastomers. However, a low-current output signal (typically in the range of nano-amperes) and bulky and expensive measurement equipment for useful signal acquisition inhibits their wearability. Herein, through a finite element analysis we establish the design rules for a highly sensitive piezo-resistive pressure sensor with an output that is high enough to be detectable by simple and inexpensive circuits and therefore ensure wearability. We also show that, out of four frequently reported micro-feature shapes in micro-patterned piezo-resistive sensors, the micro-dome and micro-pyramid yield the highest sensitivity. Furthermore, investigations of different conductivity values of micro-patterned elastomers found that coating the elastomer with a conductive material (usually metallic) leads to higher current response when compared to composited conductive elastomers. Finally, the geometric parameters and spatial configurations of micro-pyramid design of piezo-resistive sensors were optimized. The results show that an enhanced sensitivity and higher current output is achieved by the lower spatial density configuration of three micro-features per millimeter length, a smaller feature size of around 100 μm, and a 60–50 degrees pyramid angle.

## 1. Introduction

Over the past decade, research in the field of ultra-sensitive pressure sensors have seen an upsurge [1,2,3,4,5]. This is due to their potential applications in wearable and flexible electronic sensors for motion detection, biomedical monitoring, human–machine interaction, and also artificial intelligence-assisted tactile sensing [6,7,8]. Depending on the eventual application, the pressure ranges in which the sensor operates are categorized into four regimes: ultra-low pressure (<1 Pa), subtle-pressure (1 Pa–1 kPa), low-pressure (1–10 kPa), and lastly medium-pressure (10–100 kPa) [9,10]. Of all these categories, significant attention has been paid to the subtle-pressure regime because of its importance in development of electronic skin (e-skin), touch screen devices, and the non-invasive detection of weak human physiological signals such as blood pressure and pulse wave detection on the wrist. Different successful schemes such as piezo-resistive [7,11,12,13], capacitive [10,14,15,16,17], piezoelectric [18,19,20], and triboelectric [21,22] have been reported. Specifically, piezo-resistive sensors have drawn much attention because of their fast response, broad detection range, simple structure, and the simplicity of their signal measuring system.

In recent years, various structures and sensing materials have been proposed to achieve highly sensitive piezo-resistive pressure sensors, including paper based methods by Pataniya et al., that are promising [3,5,13]. One of the popular sensor platforms is based on elastomers, such as conductive polymeric films [7,11,23], or composites with distinct structural schemes such as micro-pyramids [12,24,25,26], micro-domes [12,27,28], micro-pillars [28], and micro-cones. However, the lack of design rules for the fabrication of these microstructures inhibits the achievement of the highest sensitivity in a lowest footprint area. Although there have been successful piezo-resistive pressure sensors, achieving high levels of the sensitivity to detect human pulse wave, sound wave, or subtle pressure changes caused by object manipulation are still challenging. In almost all cases, the output of the sensors when measuring weak physiological signals, such as a pulse from the wrist, are in range of nA [29,30]. That is why so far, researchers need to utilize source meters or equivalent signal acquisition devices, which are typically desktop-sized devices, to generate a useful response of the piezo-resistive sensors for these applications. The usage of such devices as an overlooked necessary amendment of these pressure sensors is against the wearability claim, which is frequently made [10,31,32,33,34,35]. Therefore, it is essential to optimize the microstructure’s geometric parameters and spatial configuration to achieve as high a sensitivity and signal level as possible, so that the dependability of the sensor response read-out on complex and non-portable measuring devices, such as source meters, is eliminated. A higher signal level is especially desired since it increases the signal-to-noise ratio, which allows for an easy signal acquisition for the sensor with simple electrical circuitry. A high enough signal-to-noise ratio also allows straightforward signal amplification, which is a necessary step for the detection of weak physiological human signals (e.g., pulse waveform from the wrist) using miniaturized and inexpensive circuits that can be integrated to wearable platforms.

So far, several attempts of designing and optimization via the modelling of piezo-resistive pressure sensors have been made; however, they are all limited to the detection of gas pressure by incorporating a sensitive diaphragm [36,37,38,39]. Currently, there has been no successful effort in the design optimization of contact piezo-resistive pressure sensors suitable for mounting on the skin to detect weak human physiological signals via a completely wearable setup. Therefore, in this study, we used a finite element method (FEM) analysis to study the optimized microstructure shape, spatial configuration, and sensing material characteristics. Three dimensional (3D) simulations have been utilized for a realistic analysis of the effects that various micro-feature shapes have on piezo-resistive sensor sensitivity. Furthermore, we investigated assigning different conductivity values to micropatterned elastomer to study the effects on the sensor’s current output level and sensitivity. Finally, the design parameters of a micro-patterned sensor (e.g., micro-pyramid) including spatial number density, size, and angle were optimized in order to achieve the maximum sensitivity in the same footprint area.

## 2. Simulation

A computational model of a piezo-resistive pressure sensor to simulate electrical output signal while the sensor undergoes compression was developed using COMSOL Multiphysics in order to solve the controlling partial differential equations by a finite element technique. The partial differential equations of physics are usually formulated either in a spatial coordinate system, with coordinate axes fixed in space, or in a material coordinate system following the material as it deforms. The former is often referred to as an Eulerian formulation, while the latter is a Lagrangian formulation.

In this study, a method called the Arbitrary Lagrangian–Eulerian (ALE) has been used for the following reason: because of the deformation associated with the micro-features during the sensor’s operation, the map from mesh coordinates to spatial coordinates might get progressively ill-conditioned (a drawback of Lagrangian method). To prevent this, a remeshing operation (stopping the simulation and deleting the previous mesh and generating a new one) is needed to map all the quantities to a regular-shaped new mesh. The method that allows rewriting the physics equations on a freely moving mesh leads to the ALE method. ALE represents an intermediate between Eulerian and Lagrangian depending on the characteristics of the study, combining the best features of both methods.

**Figure 1:**
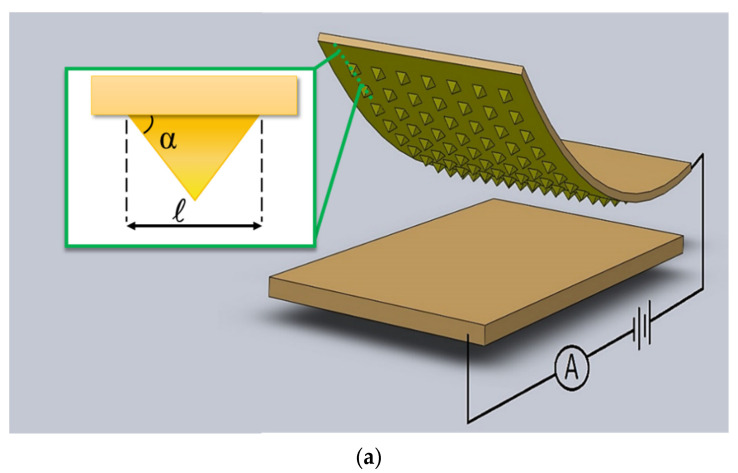

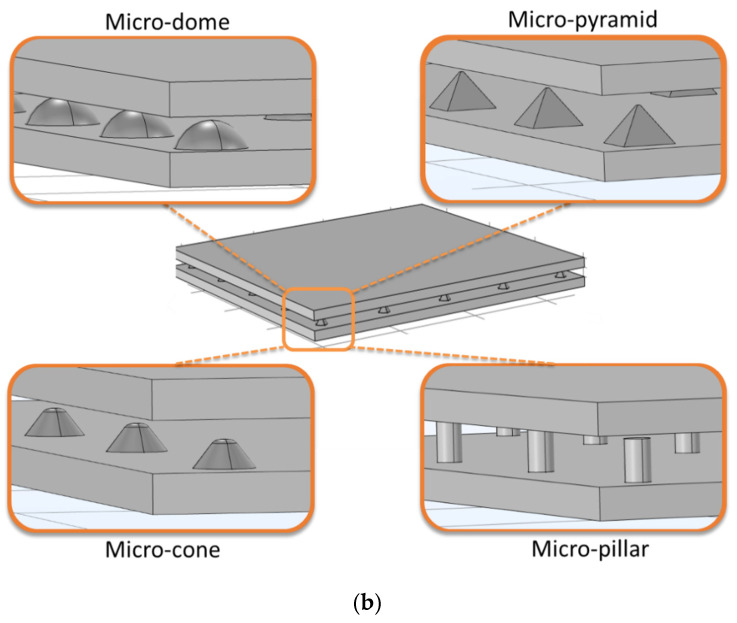
Piezo-resistive sensor. (**a**) Schematic of a micro-pyramid piezo-resistive sensor showing parameters such as pyramid angle “α” and pyramid base size “*ℓ*”. (**b**) Different proposed micro-feature shapes for a three dimensional 1.8 mm × 1.8 mm piezo-resistive sensor.

We considered steady state modeling to analyze the maximum signal level that the sensor is capable of outputting. A 3D-computational model of a square shaped pressure sensor was proposed. Basically, this modeling was proposed to simulate and study the micro-feature deformations due to pressure ranges experienced similarly to the pulse wave from the human wrist. This pressure was reported to range from 1 to 10 kPa depending on the test-subject’s characteristics [10,26]. The schematic design of a pressure sensor is shown in Figure 1a. The sensor consists of a flexible hyperelastic micro-patterned layer and a conductive current collector layer both facing each other. Various micro-feature shapes, namely the micro-pyramid, micro-cone, micro-dome, and micro-pillar, were selected for the simulation based on the frequently reported microfabricated pressure sensors (Figure 1b). 3D simulations were run to study the influence of various micro-feature shapes on the sensor’s sensitivity (which is defined as relative current change vs. applied pressure). In addition, the dependence of the sensor response on the conductivity of the flexible polymeric layer and micro-feature geometric dimensions were evaluated using 3D and 2D modeling.

Structural contact in this study, which by default dictates geometric nonlinearity, was simulated using the Augmented Lagrangian method. This setting leads to more accurate results compared to the default penalty method. Also here, E_char_ (generally equal to the young’s modulus of the destination) was increased (×100) to account for strain nonlinearity of the polymer. Finally, a preset penalty factor tuned for stability was employed. Problems incorporating large overclosure (gap), such as the present study, where the boundaries at initial steps move toward each other until they establish contact require tuning for stability.

For 3D simulations, free tetrahedral mesh type was chosen with calibration for general physics in “extremely fine” size. The type of the mesh was chosen based on a mesh quality test (measuring skewness to be near 1 which corresponds to regular shape) in order to avoid inverted mesh elements. The optimum size of the mesh was chosen based on the outcome of the convergence test. The general method for such a test was increasing the number of degrees of freedom (DOFs) associated with “extremely fine” mesh size setting to 1000% of its original value. If the results of the new study (current in amperes) varied more than 5% compared to the previous study, the convergence test would fail, and a new study with smaller mesh size would be proposed until it passed this convergence test.

Geometric parameters such as angle, base size, and spatial configuration for one of the frequently reported shapes in the literature (i.e., micro-pyramid), was chosen to optimize the micro-feature parameters. It should be noted that results obtained from such simulations were not exclusive to the micro-pyramid and could also be expanded to other micro-feature shapes. Specifically, 2D modeling was used to find the optimum angle (*α*), base size (*ℓ*) (Figure 1a), and micro-feature number density (the number of micro-features per unit length) of the pressure sensor in order to achieve the most sensitivity.

The present study used COMSOL Multiphysics to deduct the design rules for a sensitive and wearable micro-fabricated piezo-resistive pressure sensor. COMSOL Electric Currents (ec) interface from the branch AC/DC > Electric Currents (ec), coupled with Solid Mechanics module, were used to solve the differential form of Maxwell’s equations considering simulation parameters reported in Table 1.

### 2.1. Equations

In the FEM tool used in this study, models are described in terms of the partial differential equations for the underlying physical laws. Conservation of charge in the volume of the sensor dictates the rate at which the charge flows in/out of the sensor must be equal to the rate it increase/decreases inside the volume. This notion is mathematically expressed by equation of continuity as:

(1) $$\nabla \cdot J=Q_{j,v}$$

where J is the current density, and Q_j,v_ is electric charge density’s 2nd order matrix. Also, the current density is calculated by equation below,

(2) $$J=\sigma E+{\;J}_{e}$$

where σ is electric conductivity of the material (of sensing layer and the current collector layer), and E is the electric field strength, and J_e_ is the current density of an externally generated current. As seen below, electric field strength (E) is a function of the electrical potential (V):

(3) E=−∇V

These equations are solved by finite element method with numerically stable edge element discretization combined with solution of sparse equation system [43].

### 2.2. Modelling Setup

The proposed sensor setup is made up of two layers. One layer is a flat conductive substrate as the current collector and the other layer is an elastomeric PDMS substrate studded with micro-features. First, we considered an array consisting of 5 × 5 of equally spaced micro-features, each having a footprint area equivalent to 100 × 100 μm^2^. The total size of the sensor was designed to be 1.8 × 1.8 mm^2^ in order to realistically model a pressure sensor that overlies on top of the radial artery, which was reported to have diameter of about 2.3 mm in human wrist area [44].The micro-featured layer is placed facing the current collector layer so that application of an external force causes the micro-features to deform and lead to an increase of the contact area between the layers (Figure 1a). In this simulation, the formerly mentioned external forces are applied in such a way that each layer gets closer to the other 0.5 micrometer per step. In a series of 24 steps, the layers are increasingly pushed against each other causing measurable deformations on the tip of the micro-features (i.e., contact point between the two layers). This deformation leads to decreases in electrical resistance between the layers. Now, if an electrical potential difference is applied between these two layers, the passing current bridging the layers varies depending on the amount of contact area change which is caused by externally applied pressure.

In the first series of simulation modeling, the effect of different micro-feature shapes on current change versus applied pressure (sensor response) has been studied. Specifically, the elastomeric layer is studded with micro-feature shapes of dome, pillar, pyramid, and cone as shown in Figure 1b. Secondly, the influence of the sensing material’s conductivity on the sensitivity and the level of the current have been studied. To study the electrical conductivity of various available sensing materials, such as CNT incorporated PDMS [45] to Gold coating [41], different conductivity values has been assigned to the elastomeric layer (1 S/m to 10^5^ S/m). And finally, in the third series of simulations which was done in 2D, a micro-feature shape, namely the micro-pyramid, was chosen and its geometric dimensions and spatial arrangements were optimized. The relevant materials data of the simulation setup is summarized in the Table 1.

### 2.3. Modelling Assumptions and Boundary Conditions

Several assumptions that have been made in this simulation are as follows. 3D simulations have been utilized for realistic analysis of the effects that various micro-feature shapes have on sensor response. However, due to the high volume of calculations, parameters consisting of angle, base size, and number density of micro-pyramid design were optimized by 2D simulations. The effect of the temperature and humidity variation of the conductivity is assumed to be insignificant. Moreover, the materials properties assigned to each layer are uniform throughout that layer, and there are no localized variations. Rather than assuming that a coat of conductive material is deposited on micro-features in the flexible sensing layer, the whole layer is considered to be conductive (with different values of conductivity to represent different values associated with the available sensing materials). The micro-patterned flexible layer modelled to have the conductivity of a sputtered 200 nm thick gold coating (experimentally verified to be 0.1 times of the pure gold’s conductivity). The compressive force is applied normally and uniformly to the elastomeric layer to compress it against current collector layer, which is rigid and fixed in space. This flexible layer is modeled to exhibit hyperelastic behavior, while nearly incompressible according to Mooney–Rivlin material model [46]. Finally, an electrical potential difference of 1 volt is applied between the two layers by assigning ground to the flexible layer and +1 V to the current collector layer for generation of passing current between the layers (Figure 1a).

## 3. Results and Discussion

### 3.1. Influence of Micro-Feature Shape

**Figure 2:**
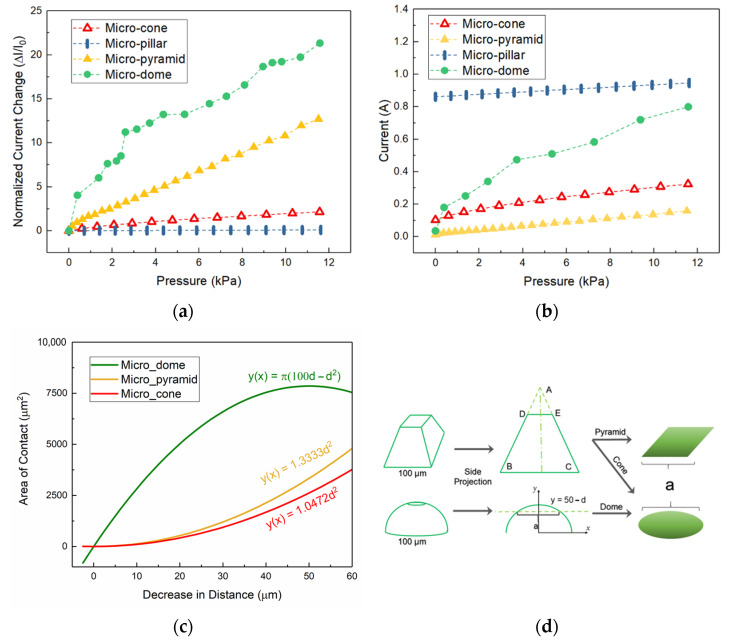
Simulation results for three dimensional pressure sensor. (**a**) Change in relative current as a function of normal pressure. (**b**) Passing current as a function of normal applied pressure. (**c**) Plot of area of contact between the two layers of the sensor as a function of decrease in layer spacing distance. (**d**) Schematic of a pyramid and cone that undergoes compression and dependence of contact area on decrease in inter-layer spacing distance.

Figure 2 shows the result of the simulations studying the effect of different micro-feature shapes on the sensitivity of the sensor when the footprint area of each micro-feature and the distance to the neighboring micro-feature remains constant (100×100 μm^2^ and 3 mm^−1^ respectively). Initially, slight pressure between the micropatterned layer and the current collector layer establishes a minimal contact area that allows electrical contact between the layers causing an initial current response (I_0_). As the pressure gradually increases on the sensor the localized deformations lead to increased contact area between the two layers. Depending on the shape of the micro-features in the elastomeric layer, the rate at which the contact area increases with applied pressure varies. In principle, due to geometrical differences associated with different shapes the linearity, sensitivity, and the current level response of the sensor can vary.

Figure 2a,b shows the results of 3D modelling of four different sensors with the size but distinct micro-feature shapes. As the pressure pushing the layers against each other increases from 0 to 12 kPa, the response from micro-dome shows the highest slope which translates to highest sensitivity among all the proposed shapes. Furthermore, the frequently reported [25,26] micro-pyramid design also displays a good sensitivity in the range mentioned. Other designs namely micro-cone and micro-pillar show lower relative sensitivities, however, the latter shows a higher initial current response which can be useful in applications such as switch type pressure sensors with digital mode of operation. Linearity in a sensor is desired because of both the mathematic simplicity that allows for sensor’s response prediction, and also enabling the detection of an irregular sensor response. Although micro-domes offer excellent sensitivity, they do not offer linear response. On the other hand, micro-pyramid arrays exhibit clear linearity with acceptable sensitivity. Other micro-feature shapes also show linearity however they lack the high sensitivity of micro-pyramid design (Figure 2b).

**Figure 3:**
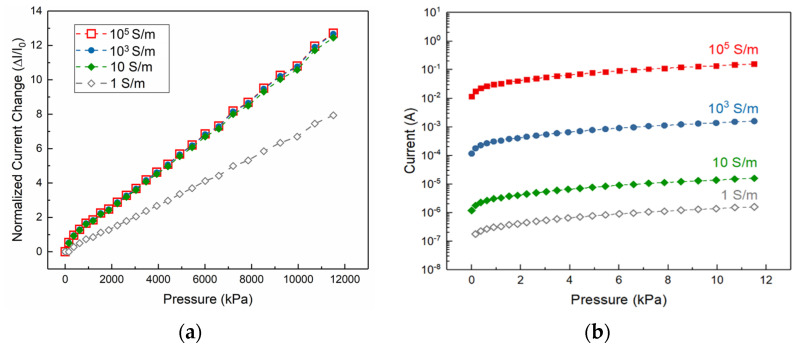
(**a**) Change in relative current as a function of applied normal pressure. (**b**) Current response of the sensor with different conductivity values.

To further explain why micro-feature shapes have the abovementioned effects on the sensor’s response, an analytical reasoning is provided as follows. If we consider micro-pyramid as an example of a micro-feature in micro-patterned piezo-resistive sensors, the Thales theorem describes the relationship between the shape and the contact area change when the sensor undergoes compression as the result of its operation (Figure 3). Since at low pressure ranges (0–12 kPa) compression force only causes rather minimal deformations at the tip of the micro-feature it can be deduced that the side projection of a pyramid is deformed simply from a triangle into a trapezoid (Figure 3a). As a result, Thales theorem Equation (4) can be applied to the initial and the final shapes after the compression to find a mathematical relation governing how length “*a*” (represents the length of the contact area) changes when the sensor is deformed under compression (Figure 2d). Thus, considering the Thales theorem, Equation (4) and Figure 2d, the relationship between “*a*” (the width of the flattened tip area) and “*d*” (displacement of top layer against elastomeric layer) can be mathematically expressed as Equation (5).

(4) $$\frac{DE}{BC}=\frac{AD}{AB}=\frac{AE}{AC}$$

(5) $$\frac{\frac{1}{2}\times a}{\frac{1}{2}\times \ell }=\frac{d}{\frac{1}{2}\times \ell \times \tan\alpha }$$

It can be seen that for a pyramid with a chosen angle of 60 degrees (*α*), the relationship between “*a*” and “*d*” is established by trigonometry. And from the Figure 2d, it can be perceived that the contact area between the layers is in form of a square whose sides have length of “*a*”. Therefore, simply the contact area of a pyramid micro-feature (S_p_) is given by Equation (6). Similarly, for the case of a micro-cone studded sensor (also with an angle of 60 degrees), since the side projection of the micro-cone is the same as the micro-pyramid, the relationship between “*a*” and “*d*” does not change. However, the micro-cone forms a circular contact area whose diameter equals to “*a*”. This surface area is given by Equation (7).

(6) $$S_{p}=1.3333\;d^{2}$$

(7) $$S_{c}=\frac{\pi }{4}a^{2}=1.0472\;d^{2}$$

On the other hand, in the case of micro-dome structures, considering the side projection of a dome (Figure 2d) and forming a system of equations as follows Equation (8). The length “*a*” is basically the distance between the two points of intersection of the aforementioned lines which is expressed by Equation (9). Finally, using the value obtained for “*a*” then the contact area of the dome which is in form of a circle is calculated by Equation (10).

(8) $$\left\{ \begin{array}{c} x^{2}+y^{2}=50^{2}\\ y=50-d \end{array}\right.$$

(9) $$a=2\sqrt{100d-d^{2}}$$

(10) $$S_{d}=\pi \left(100d-d^{2}\right)$$

It should be also noted that since we have assumed no lateral flow of material during compression, deformation of micro-pillar causes no contact area increase. The Equation (9) modest change in current response of the sensor in the simulation results is due to the lateral material flow and increase of contact pressure that leads to higher inter-layer conductivity because of microscopic surface roughness flattening. Therefore, the developed analytical relationship is in accordance with simulation results.

Utilizing the above developed analytical equations, the change of contact area vs. decrease of layer spacing for each micro-feature shape is plotted in Figure 2c. The observed trends in this plot are in accordance with the current responses seen in the COMSOL simulation results. Here, for pressure ranges of 0–12 kPa, which corresponds to 0–20 μm of layer spacing decrease, the micro-dome’s results show the steepest slope (i.e., the highest rate at which the surface area changes when the layers get closer together). Moreover, the micro-pyramid design results show a higher slope than micro-cone’s which is due to the geometry of the shape as discussed before.

### 3.2. The Effect of the Conductivity on the Sensor’s Response

Here, the sensor’s response is investigated in terms of sensitivity and the passing current level with various elastomeric layer conductivities when the shape, geometrical parameters and spatial configuration of the micro-feature remains constant (pyramid, 100×100 μm^2^ and 3 mm^−1^ respectively). The simulation results show that the sensitivity of the sensor saturates at the elastomeric layer conductivity of 10 S/m and becomes independent of it. However, if the conductivity value falls below 1 S/m which is in range of conductive polymers such as carbon nano tube (CNT) infused PDMS [45], the sensitivity deteriorates. At these conductivity values the high resistance of the elastomeric layer serves as a limit to the passing current between the layers and thus reducing the overall sensitivity of the sensor. For this reason, in order to achieve high sensitivity in piezo-resistive sensors, it is recommended that elastomeric layer be coated with a highly conductive layer like gold or as an example other novel conductive materials such as Zirconium nitride (~2 $\times 10^{6}$ S/m [47]) with conductivity values higher than 10 S/m. In most cases, compositing the elastomeric layer with conductive materials leads to polymers that are not conductive enough which would adversely affect the sensitivity of the pressure sensor.

One of the most important characteristic of the piezo-resistive pressure sensor is the magnitude of output signal. In this work the dependence of the sensor’s output signal level on the conductivity of the elastomeric layer (sensing layer) is investigated. Through 3D simulations of piezo-resistive sensor operation (Figure 3b), it can be found that the higher the conductivity values of the sensing material leads to higher level of passing current with the same applied voltage (3 Volts) between the layers of the sensor. In fact, a piezo-resistive sensor that is gold coated (sputtered to a thickness of 150 nm) exhibits a conductivity value of 10^5^ S/m (experimentally verified) which according to the results leads current output in range of milliamps. This is practically advantageous for two reasons. First, it allows the signal acquisition with relative simple electrical circuitry (e.g., simple and wildly accessible development boards), and second, it provides higher signal-to-noise ratio which in turn allows use of amplifiers to further facilitate the signal acquisition. While several reports of composited polymer piezo-resistive sensors exists in the literature [25,27,28], their current output signal ranges typically in nanoamperes and low microamperes in case of measuring pulse from a human wrist. This necessitates the use of relatively big, bulky, desktop-sized source meters for signal acquisition. Since most of these sensors are targeted for detection of weak physiological sensors as a wearable device, dependence of signal acquisition on bulky source meters is counter-intuitive. Therefore, use of highly conductive coating material of flexible elastomeric layer is necessary in order to achieve high level of passing current to be detectable by truly wearable signal acquisition development boards.

### 3.3. Geometric Parameters Optimization

**Figure 4:**
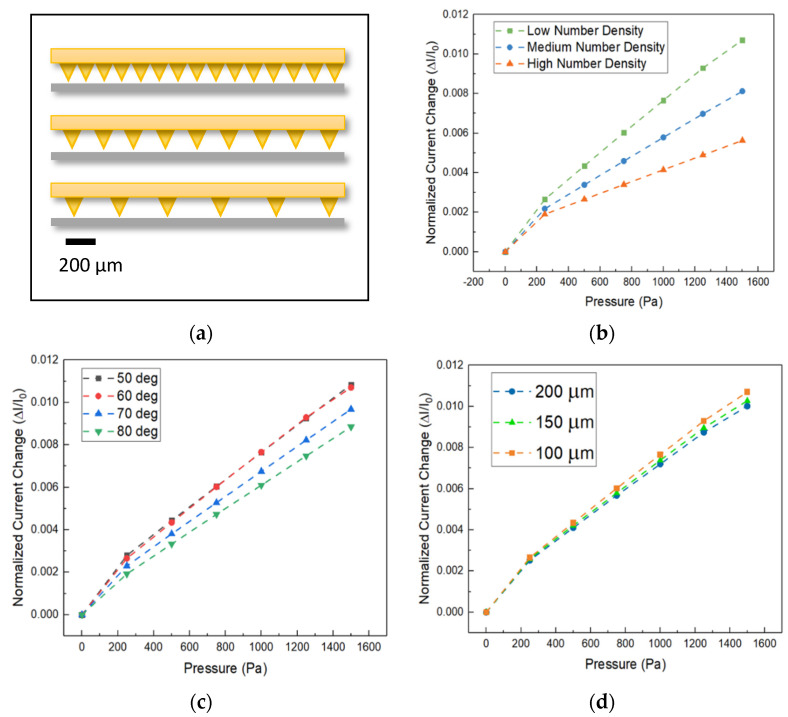
Schematic of different spatial densities under investigation (**a**). Micro-pyramid geometric parameter and configuration results in terms of relative current as a function of applied pressure for (**b**) different spatial densities, (**c**) different pyramid angles “α”, (**d**) different pyramid size “ℓ”.

In order to investigate the how the geometric parameters of a given design can influence the sensitivity, a micro-pyramid patterned sensor with conductivity value of similar to gold (10^6^ S/m), was chosen for investigation. Various external pressures in the range of 0–1500 Pa were applied to the sensor in this simulation. Parameters including micro-feature dimensions and spatial arrangements have been studied as shown in Figure 4a–d. Because of the similarities between micro-patterned pressure sensors designs, parameters optimized for one design can be easily generalized to the other micro-patterned pressure sensor shapes. Here, micro-pyramid studded sensor behavior was studied due to its similarities to other micro-patterned pressure sensors such as micro-cone, micro-dome, and micro-pillar. Therefore, sensors length of 2.1 mm with three different pyramid spatial densities consisting of low, medium, and high number density (corresponding to six, nine, and fifteen pyramids, respectively) were simulated to study the impact of micro-feature number density on sensitivity. According to the Figure 4b, low number density setup data yields to the highest slope of the fitted curve which means that this setup shows the most sensitive response among all three setups. This is due to concentration of the exerted pressure at fewer pyramids. Severer deformations at these contact points essentially mean larger contact area between the layers which in turn translates to lower resistance and higher passing current.

To quantitatively compare the sensitivity arisen form setups with different pyramid angles at the same sensor length of 2.1 mm, micro-pyramid designs with angles (shown as “*α*” in Figure 1a) between 80 to 50 degrees have been studied in Figure 4c. The results depict that the typically pyramids with angles of 50 to 60 degrees show relatively a more sensitive response. The reason for this lies in a balance between the contact area growth rate (highest growth rate corresponds to 50 degrees according to the Equation (6) and the amount of localized pressure at contact areas between the layers. It should be noted that in order to achieve high contact area growth rate, the localized pressure has to be high enough to enable the deformation. The highest localized pressure is typically experienced in structures with low contact area growth rate (i.e., 80 degrees). Therefore, it is logical to achieve the highest sensitivity with a sensor that is studded with 50 to 60-degree angle pyramids in which case both factors have medium and balanced influence. This is in turn in accordance with the aforementioned simulation results. Furthermore, an easier way to manipulate the design of a micro-feature studded sensor, rather than changing the angle, is to change the size of the micro-features themselves. Practically this is done by changing the microfabrication mask. To study this, simulations were conducted by changing the size of micro-features (depicted as “*ℓ*” in Figure 1a) with three base sizes 100, 150, 200 µm all with previously determined sensitive configurations which are 60-degree pyramid angle and low number spatial density. As shown in Figure 4d, the normalized current change (i.e., sensitivity) increases as the sensor is compressed under external pressure. All three configurations show linear growth behavior; however, as the feature size gets smaller, namely 100 µm, the slope of the line increases. The higher slope of the fitted lines means higher sensitivity. This is attributed to increasing of the localized pressure because of the smaller contact areas associated with smaller feature sizes.

Overall out of the three above-mentioned geometric parameters, spatial number density of micro-features represents the most influential factor on sensitivity of the sensor since localized pressure experienced at the contact areas, strongly depends on the total number of points of contact between the layers. The fewer the number of contact areas (i.e., lower spatial number density), the higher localized pressure and therefore more deformation and lower electrical resistance between layers. While dimensional parameters such as base size and angle of pyramid show weaker influence on the sensitivity of the sensor, they also have to be taken into consideration in designing of highly sensitive micro-patterned piezo-resistive pressure sensors. Thus based on the findings of this work, a micro-patterned piezo-resistive pressure sensor for arterial pulse monitoring in contact with skin can achieve potentially higher sensitivity and signal strength if the micro-features are in the shape of domes or pyramids and they are patterned with number density of 3 mm^−1^, feature size of 100 μm, and angle of 50° < α < 60° (in case of pyramid shape). Also, elastomer layer has to at least have the conductivity of 10 S/m to ensure that the sensitivity does not deteriorate due to lack of conductivity. Nonetheless, as the conductivity of the elastomeric layer is enhanced to approach the conductivity of gold, the sensor signal output becomes stronger (i.e., higher current) which is favorable for detection of the signal with simple and inexpensive electrical circuits tailored for wearable applications.

## 4. Conclusions

In this paper, a series 2D and 3D simulations were conducted to compare the effect of different shaped micro-features on the sensitivity and signal level of a piezo-resistive sensor. We have shown that sensors with arrays of micro-domes and micro-pyramids show higher sensitivity in comparison to micro-cone and micro-pillar studded ones. Moreover, simulations on assigning different values of conductivity provided insights that, in conductivity values of the sensing layer similar to that of the gold coating, the sensor achieves a high signal level response. However, if the conductivity of the sensing layer is similar to typical conductive polymers’, the sensitivity suffers and the current response is so low that inhibits the practical use of the sensor due to low signal-to-noise ratio. Finally, in a series of 2D simulations it is shown that lower spatial number density in arrays of micro-features, and smaller base size leads to higher overall sensitivity of the micro-patterned piezo-resistive sensor.

## Figures and Tables

**Table 1:** Simulation parameters.

Parameter	Micro-Patterned Layer	Current Collector Layer	References
Feature Angle (*α*)	57.4 degrees	N/A	[40]
Feature Base Size (*ℓ*)	100 µm	N/A	N/A
Feature Spacing	300 µm	N/A	N/A
Array	5 × 5 (low number density setup)	N/A	N/A
Footprint	1.8 × 1.8 mm^2^	1.8 × 1.8 mm^2^	N/A
Conductivity	1 $\times 10^{5}$ S/m	46$\times 10^{6}$ S/m	[41]
Young’s modulus	750 kPa	70 GPa	[41,42]
Poisson’s ratio	0.49	0.44	[41,42]
Density	970 kg/m^3^	19,300 kg/m^3^	[41,42]
Relative Permittivity	2.75	1	[41,42]

## Data Availability

The datasets generated during and/or analyzed during the current study are available in the Data for article: Design rules for wearable repository, https://drive.google.com/drive/folders/1QCR88TUgAIagCl3VK5_poOsU7ujR1Dmc?usp=sharing (1 May 2022).
